# Outcomes of the transformation of follicular lymphoma to diffuse large B‐cell lymphoma in the rituximab era: A population‐based study

**DOI:** 10.1002/cam4.7120

**Published:** 2024-04-17

**Authors:** Wenshuai Zheng, Mingjuan Liu, Lixun Guan, Shenyu Wang

**Affiliations:** ^1^ Department of Hematology Hainan Hospital of Chinese PLA General Hospital Sanya China; ^2^ Senior Department of Hematology Fifth Medical Center of Chinese PLA General Hospital Beijing China

**Keywords:** diffuse large B‐cell lymphoma, follicular lymphoma, population‐based, SEER, survival, transformation

## Abstract

**Background:**

Histological transformation (HT) to diffuse large B‐cell lymphoma (DLBCL) is a common complication of follicular lymphoma (FL) and is usually associated with a dismal outcome. However, the survival rate of these patients has improved over the last 20 years with the introduction of rituximab. This study aimed to access the outcome of transformation to DLBCL (t‐DLBCL) from FL in a retrospective series that began after the widespread use of rituximab use. In addition, we also compared survival between t‐DLBCL and primary DLBCL (p‐DLBCL) in the same timeframe.

**Methods:**

We utilized the Surveillance, Epidemiology, and End Results (SEER) database to identify patients with primary FL and patients with p‐DLBCL between 2000 and 2020. Patients who had a subsequent diagnosis of DLBCL at least 2 months after FL diagnosis were identified as t‐DLBCL.

**Results:**

Finally, we identified 50,332 FL and 95,933 p‐DLBCL. With a median follow‐up of 119 months, 1631 patients developed t‐DLBCL. The median time from FL diagnosis to t‐DLBCL was approximately 4 years. The post‐transformation survival (PTS) rate at 5 years was 49.6%, with a median PTS of 56 months. Older age, advanced stage, and early transformation were associated with worse PTS. Furthermore, t‐DLBCL receiving chemotherapy or combined modality as initial therapy before HT was also associated with worse PTS, while the result was inverse when taking the impact of initial management strategy at HT into account. Taking t‐DLBCL and p‐DLBCL as a whole, comparable survival was observed between p‐DLBCL and t‐DLBCL receiving radiation or watch‐and‐wait as initial therapy prior to HT.

**Conclusion:**

The outcome of t‐DLBCL in the rituximab era was better than historical series before the rituximab era. Due to the good prognosis, we did not recommend autologous stem cell transplantation for t‐DLBCL receiving watch‐and‐wait or radiation as initial therapy before HT.

## 1 INTRODUCTION

Follicular lymphoma (FL) is the most common subtype of indolent non‐Hodgkin lymphoma. It is characterized by an indolent clinical course accompanied by a continuous pattern of relapses. In recent decades, the survival of FL has improved significantly due to the extensive use of anti‐CD20 monoclonal antibody rituximab, approved for the treatment of FL in late 1997, in standard induction and maintenance regimens,^1^, ^2^, ^3^, ^4^ Nevertheless, histological transformation (HT), the most common diffuse large B‐cell lymphoma (DLBCL), still remains a critical event in the natural history of FL. According to studies conducted after the introduction of rituximab,^5^, ^6^, ^7^, ^8^, ^9^ the prognosis of transformed FL (t‐FL), previously considered very poor with a median survival of approximately 1 year,^10^, ^11^, ^12^, ^13^, ^14^ has improved in the rituximab era. Nonetheless, post‐transformation survival (PTS) varies widely between studies in the rituximab era, with reported 5‐year PTS ranging from 41% to 75%.^5^, ^6^, ^7^, ^8^, ^9^ Therefore, we conducted this population‐based analysis based on Surveillance, Epidemiology, and End Results (SEER) database to further clarify the outcome of transformation to DLBCL from FL (t‐DLBCL) in the rituximab era. In addition, we also compared the survival outcome between t‐DLBCL and primary DLBCL (p‐DLBCL), which has been rarely reported.

## 2 PATIENTS AND METHODS

### 2.1 Patients

Data of this study were obtained from the National Cancer Institute's Surveillance, Epidemiology, and End Results (SEER) database, which is an authoritative source of population‐based cancer statistics in the United States (US) and currently covers approximately ~30% of the US population.^15^ Our analysis was limited to the SEER 17 registry database, released in April 2023 based on the November 2022 submission.

In the SEER cancer registry, all FL cases were identified using the third edition of the International Classification of Diseases for Oncology (ICD‐O‐3) code 9690, 9691, 9695, and 9698, and all p‐DLBCL cases were identified using the ICD‐O‐3 code 9680. Patients who had a subsequent diagnosis of DLBCL at least 2 months after FL diagnosis were identified as t‐DLBCL, which can avoid ascertainment bias. The exclusion criteria were: (1) FL or p‐DLBCL diagnosis confirmed only by autopsy or death certificate; (2) age <18 years; and (3) patients who were not the primary malignancy. Histology was divided into grades 1, 2, 3, and unknown according to the World Health Organization histological classification. The stage was divided into early stage (stage I–II), advanced stage (III/IV) and unknown. We use summary stage for lymphoma to determine the exact stage. Age was divided into four age groups: 18–59, 60–69, 70–79, and ≥80 years. Involvement of extranodal sites was divided into “yes” and “no.” Radiation therapy (RT) and chemotherapy (CT) were classified as “yes” and “no/unknown.” The SEER database records first‐course of therapy data, which is defined as a treatment plan initiated within 12 months of diagnosis. Second‐line therapies are not recorded. Therefore, patients receiving RT and CT simultaneously were defined as patients receiving combined modality (CM) as initial treatment strategy, and patients receiving neither RT nor CT were defined as patients receiving watch and wait (WW) as an initial treatment strategy.

### 2.2 Statistical analysis

Overall survival (OS) for FL and p‐DLBCL was defined as the date of diagnosis to the date of death (all causes) or last follow‐up. Post‐transformation survival (PTS) was defined as the date of HT to the date of death (all causes) or last follow‐up. The latency period was defined as the date of FL diagnosis to the date of HT. In exploratory analyses, we assessed early versus late transformation using an a priori defined cutoff of 18 months. Survival curves were reported using Kaplan–Meier estimates, and statistical comparisons between curves were peformed using Log‐rank test. To evaluate the influence of various covariates on survival, we used Cox proportional hazards regression model, including all significant variables in the univariate analysis (*p* < 0.05), to calculate hazard ratios (HR) and 95% confidence intervals (CI).

Data were extracted using SEER*Stat (version 8.4.1.2). Statistical analysis was conducted using R 4.2.3 software (R Development Core Team). All *p* values were calculated as two‐sided, and *p* values less than 0.05 were considered statistically significant.

## 3 RESULTS

### 3.1 Patients' characteristics of t‐DLBCL

Finally, 50,332 patients with FL and 95,933 patients with p‐DLBCL were identified during 2000–2020. Of the patients with FL, 48,868 patients survived ≥2 months, with 1631 patients (3.3%) developing t‐DLBCL after a median follow‐up of 119 months (range, 2 to 251 months). The median time from FL diagnosis to development of t‐DLBCL was 48 months (range, 2 to 233 months). Of the patients with t‐DLBCL, most were 60–69 years old 520 (31.9%) at HT, male 892 (54.7%), advanced stage 903 (55.4%) at HT, and no involvement of extranodal sites 1314 (82.2%) at HT. For the latency period, most patients developed HT after 18 months 1220 (74.8%). Regarding the initial management strategy before HT, 679 (41.6%) patients receiving WW, 149 (9.1%) patients receiving RT, 727 (44.6%) patients receiving CT, and 76 (4.7%) patients receiving CM. At HT, the proportion of patients receiving WW decreased from 41.6% to 18.9%, while the proportion patients receiving CT increased from 44.6% to 66.2%. The patients' main presenting features are summarized in Table 1.

**TABLE 1:** Univariate and multivariate analysis of post‐transformation survival for t‐DLBCL.

Clinical features	*n* (%)	Univariate analysis		Multivariate analysis	
		Hazard ratio (95% CI)	*p*	Hazard ratio (95% CI)	*p*
Age, years					
18–59	466 (28.6)	Reference		Reference	
60–69	520 (31.9)	0.978 (0.805–1.188)	0.821	0.972 (0.798–1.183)	0.774
70–79	437 (26.8)	1.497 (1.235–1.815)	<0.001	1.526 (1.258–1.852)	<0.001
≥80	208 (12.8)	2.198 (1.772–2.727)	<0.001	2.372 (1.900–2.961)	<0.001
Gender					
Female	739 (45.3)	Reference		Reference	
Male	892 (54.7)	1.131 (0.981–1.304)	0.090		
Stage before HT					
I–II	584 (35.8)	Reference		Reference	
III–IV	958 (58.7)	1.006 (0.918–1.238)	0.401		
Unknown	89 (5.5)	0.901 (0.649–1.251)	0.535		
Stage at HT					
I–II	577 (35.4)	Reference		Reference	
III–IV	903 (55.4)	1.441 (1.232–1.686)	<0.001	1.632 (1.389–1.917)	<0.001
Unknown	151 (9.3)	1.412 (1.102–1.808)	0.006	1.278 (0.988–1.653)	0.061
Grade					
1	384 (23.5)	Reference		Reference	
2	570 (34.9)	0.989 (0.819–1.195)	0.909		
3	274 (16.8)	1.200 (0.962–1.498)	0.105		
Unknown	403 (24.7)	1.039 (0.850–1.270)	0.710		
Involvement of extranodal sites before HT					
No	1424 (87.3)	Reference		Reference	
Yes	207 (12.7)	0.766 (0.610–0.962)	0.022	0.867 (0.683–1.100)	0.222
Involvement of extranodal sites at HT					
No	1341 (82.2)	Reference		Reference	
Yes	290 (17.8)	0.877 (0.726–1.058)	0.169		
Latency period					
<18 months	411 (25.2)	Reference		Reference	
≥18 months	1220 (74.8)	0.838 (0.716–0.981)	0.028	0.840 (0.716–0.986)	0.033
Initial therapy before HT					
WW	679 (41.6)	Reference		Reference	
RT	149 (9.1)	0.761 (0.567–1.022)	0.070	0.852 (0.630–1.153)	0.300
CT	727 (44.6)	1.327 (1.140–1.544)	<0.001	1.475 (1.262–1.723)	<0.001
CM	76 (4.7)	1.394 (1.012–1.920)	0.042	1.697 (1.228–2.344)	0.001
Initial therapy at HT					
WW	309 (18.9)	Reference		Reference	
RT	40 (2.5)	0.987 (0.659–1.278)	0.948	0.945 (0.625–1.427)	0.787
CT	1080 (66.2)	0.628 (0.530–0.744)	<0.001	0.613 (0.510–0.737)	<0.001
CM	202 (12.4)	0.513 (0.395–0.667)	<0.001	0.539 (0.411–0.706)	<0.001

Abbreviations: CI, confidence interval; CM, combined modality; CT, chemotherapy; HT, histological transformation; RT, radiotherapy; WW, watch and wait.

### 3.2 Impact of HT on overall survival

**FIGURE 1:**
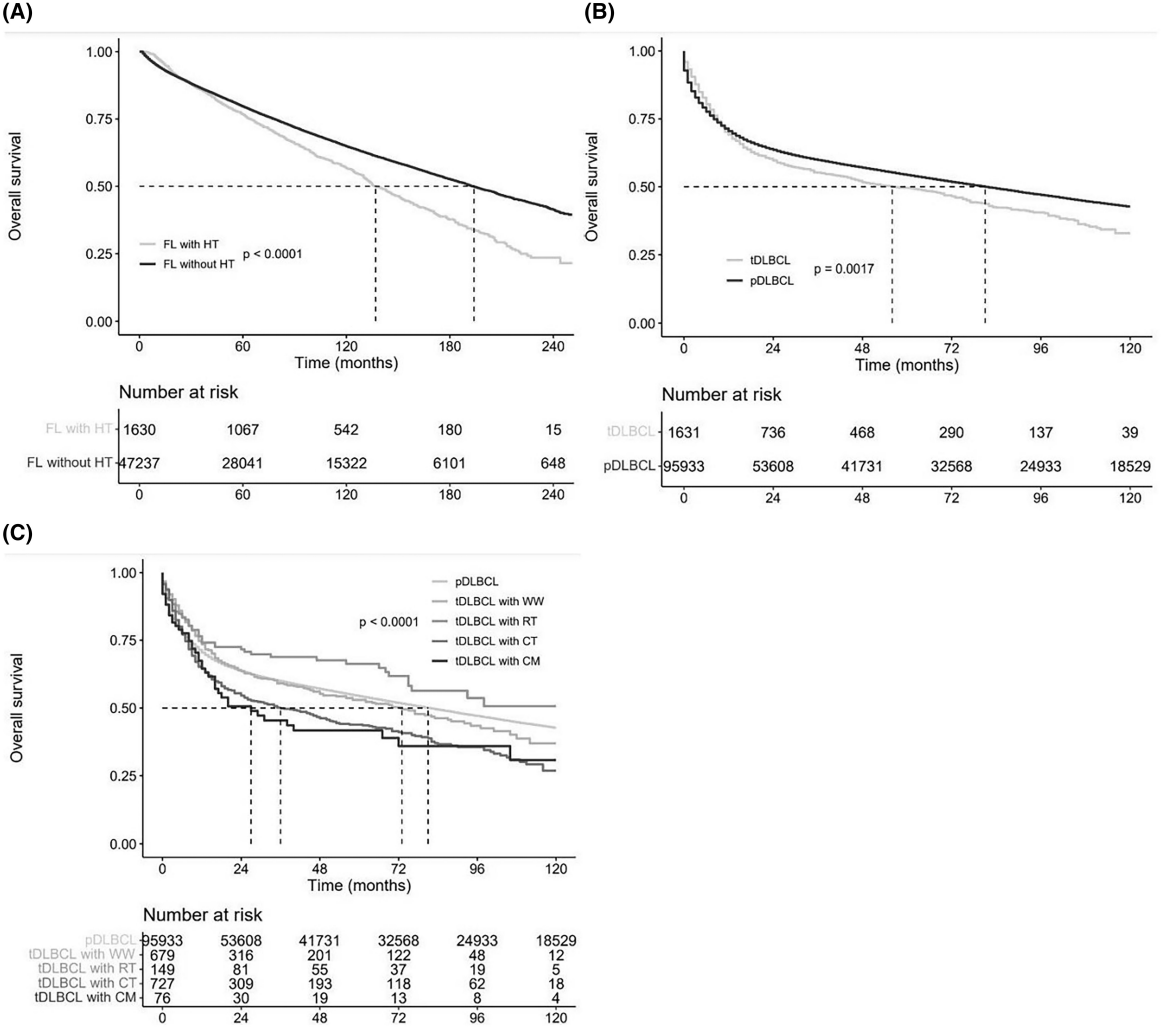
Kaplan–Meier overall survival curves. (A) FL with histological transformation (HT) versus FL without HT, (B) t‐DLBCL versus p‐DLBCL, (C) p‐DLBCL versus t‐DLBCL receiving different initial management strategies before HT.

Regarding the influence of HT on the outcome of patients with FL, Log‐rank test showed that the appearance of HT significantly decreased patient's OS (*p* < 0.001, Figure 1A). For patients with t‐DLBCL, the OS rate at 10‐year was 56.6% (95% CI, 53.9%–59.3%) with a median OS of 137 months (range, 2 to 251 months), while the OS rate at 10‐year was 64.8% (95% CI, 64.3%–65.3%) with a median OS of 194 months (range, 2 to 251 months) for patients without t‐DLBCL.

### 3.3 Outcomes of patients with t‐DLBCL

**FIGURE 2:**
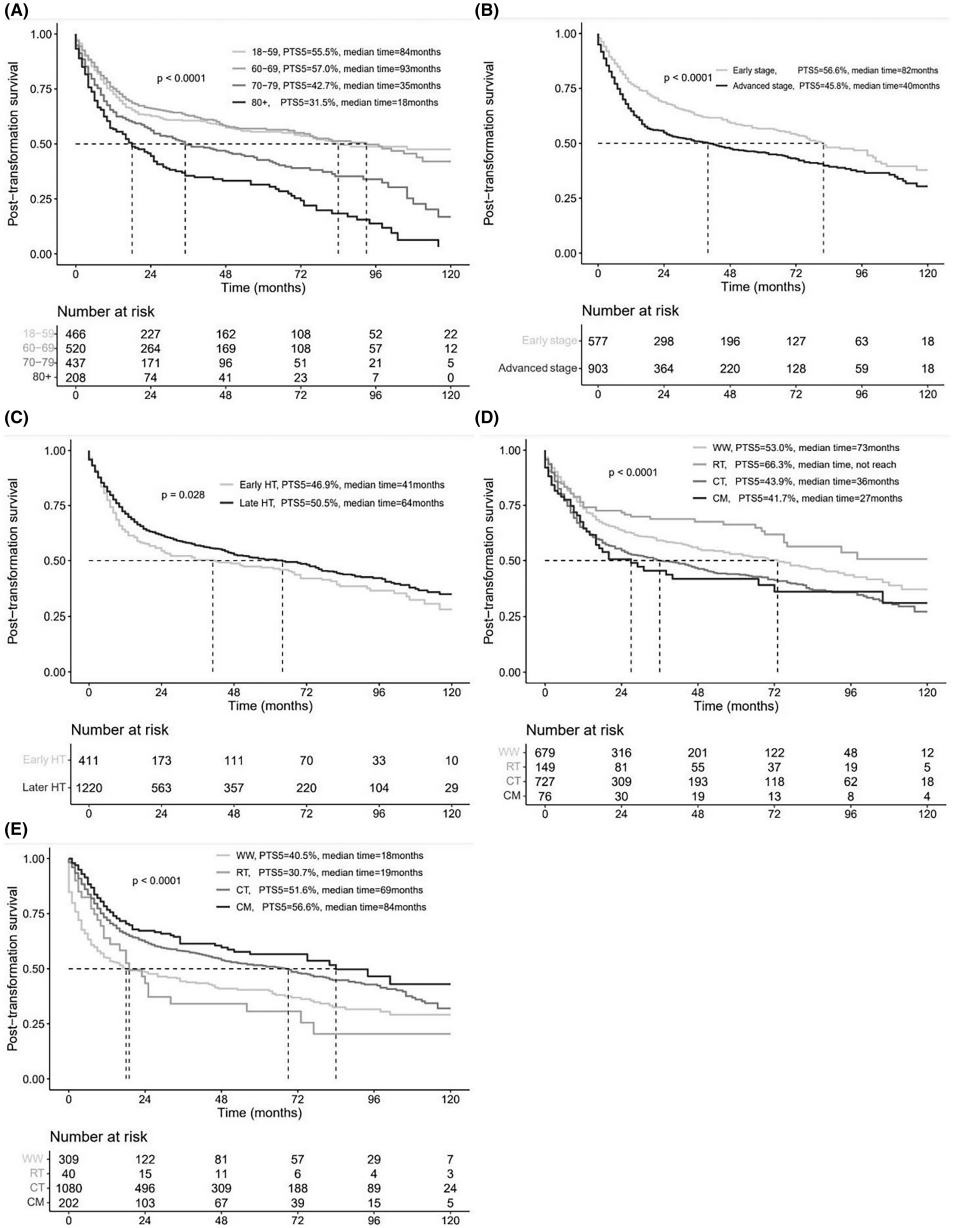
Kaplan–Meier post‐transformation survival curves for t‐DLBCL according to (A) Age at histological transformation (HT), (B) Stage at HT, (C) Latency period, (D) Initial management strategy before HT, (E) Initial management strategy at HT.

After a median follow‐up of 54 months (range, 0–222 months) after HT, 776 of 1631 patients had died. The PTS rates at 5‐year (PTS5) and 10‐year (PTS10) were 49.6% (95% CI, 46.9%–52.4%) and 33.0% (95% CI, 29.0%–37.5%), respectively, with a median PTS of 56 months (range, 0–222 months). The results of the univariate and multivariate analysis of PTS10 are summarized in Table 1, and the PTS10 curves are shown in Figure 2.

**TABLE 2:** Univariate and multivariate analysis of overall survival for DLBCL.

Clinical features	Univariate analysis	*p*	Multivariate analysis	*p*
	Hazard ratio (95% CI)		Hazard ratio (95% CI)	
p‐DLBCL versus t‐DLBCL	1.116 (1.039–1.198)	0.002	0.971 (0.937–1.006)	0.102
p‐DLBCL versus t‐DLBCL receiving WW	0.987 (0.879–1.107)	0.818		
p‐DLBCL versus t‐DLBCL receiving RT	0.762 (0.581–1.000)	0.050		
p‐DLBCL versus t‐DLBCL receiving CT	1.296 (1.173–1.432)	<0.001	1.349 (1.221–1.490)	<0.001
p‐DLBCL versus t‐DLBCL receiving CM	1.376 (1.021–1.856)	0.036	1.564 (1.160–2.109)	0.003

Abbreviations: CI, confidence interval; CM, combined modality; CT, chemotherapy; RT, radiotherapy; WW, watch and wait.

Regarding initial management strategy before HT, when taking patients receiving WW as reference, patients receiving CT (Log‐rank: *p* < 0.001; multivariate: HR = 1.475, *p* < 0.001) or CM (Log‐rank: *p* = 0.041; multivariate: HR = 1.697, *p* = 0.001) had significantly inferior PTS10, while patients receiving RT had similar PTS10 (Log‐rank: *p* = 0.063; multivariate: HR = 0.852, *p* = 0.300). When considering initial management strategy at HT, the results were inverse. When taking patients receiving WW as reference, patients receiving CT (Log‐rank: *p* < 0.001; multivariate: HR = 0.613, *p* < 0.001) or CM (Log‐rank: *p* < 0.001; multivariate: HR = 0.539, *p* < 0.001) had significantly superior PTS10, while patients receiving RT still had similar PTS10 (Log‐rank: *p* = 0.930; multivariate: HR = 0.945, *p* = 0.787).

Age at HT was also an independent prognostic factor for PTS10. Compared with patients aged 18–59 years, patients aged 70–79 years (Log‐rank: *p* < 0.001; multivariate: HR = 1.526, *p* < 0.001) or ≥ 80 years (Log‐rank: *p* < 0.001; multivariate: HR = 2.372, *p* < 0.001) had a significantly inferior PTS10, while patients aged 60–69 years had a similar PTS10 (Log‐rank: *p* = 0.760; multivariate: HR = 0.972, *p* = 0.774). In addition, the PTS10 of patients with advanced stage at HT (Log‐rank: *p* < 0.001; multivariate: HR = 1.632, *p* < 0.001) was worse than that of patients with early stage at HT. The latency period was inversely correlated with PTS10. Patients with late HT (≥18 months) after FL diagnosis (Log‐rank: *p* = 0.028; multivariate: HR = 0.840, *p* = 0.033) had significantly superior PTS10 in comparison with early HT (<18 months).

### 3.4 Comparison between t‐DLBCL and p‐DLBCL

When taking t‐DLBCL and p‐DLBCL as a whole, Log‐rank test showed that t‐DLBCL had inferior 5‐year OS (OS5) in comparison with p‐DLBCL (*p* = 0.002, Figure (1B). The OS5 was 54.3% (95% CI, 54.0%–54.7%) with a median OS of 81 months (range, 0–251 months) for patients with p‐DLBCL. However, multivariate analysis, adjusted by age, stage, extronodal sites, and initial management strategy, showed that t‐DLBCL (HR = 0.971, *p* = 0.102) was not an independent risk factor for 10‐year OS (0S10) in comparison with p‐DLBCL. Considering the PTS differences of t‐DLBCL based on the initial management strategy before HT, we performed a subgroup analysis. When taking p‐DLBCL as reference, t‐DLBCL receiving CT (Log‐rank: *p* < 0.001; multivariate: HR = 1.349, *p* < 0.001) or CM (Log‐rank: *p* = 0.034; multivariate: HR = 1.564, *p* = 0.003) had inferior OS10, while the OS10 of t‐DLBCL receiving RT (Log‐rank: *p* = 0.049; multivariate: HR = 0.987, *p* = 0.050) or receiving WW (Log‐rank: *p* = 0.870; multivariate: HR = 0.724, *p* = 0.020) were comparable to p‐DLBCL. The results of the univariate and multivariate analysis of OS10 are summarized in Table 2, and the OS10 curves are shown in Figure (1C).

## 4 DISCUSSION

t‐DLBCL is a heterogeneous disease and survival outcomes are controversial in the rituximab era, meaning more information is needed to discriminate the subgroup of patients with different prognosis. In this comprehensive retrospective series of patients with t‐DLBCL, we explored clinical characteristics affecting PTS and compared OS between t‐DLBCL and p‐DLBCL. To our knowledge, this is the largest dataset of patients with t‐DLBCL published to date.

In our study, FL with t‐DLBCL had significantly worse OS in comparison with FL without t‐DLBCL, which was similar to the results of previous studies.^5^, ^16^ These results reflected the transformation of FL from indolent to aggressive, which strengthened the necessity of performing a biopsy at the recurrence of FL. There was a significant difference in the prognosis of t‐FL, the most common DLBCL, between patients before and after the rituximab era. The outcome of patients before the rituximab era was worse with a median PTS from 0.6 to 1.7 years.^10^, ^11^, ^12^, ^13^, ^14^ In the Swiss series, in which a minority received rituximab, the median PTS improved to 2.7 years.^16^ The median PTS of our observation was 56 months, which was comparable to previous reports in the rituximab era with a median PTS nearly 5 years.^5^, ^7^, ^17^, ^18^ In addition, we also compared the outcome between p‐DLBCL and t‐DLBCL diagnosed in the same timeframe by taking t‐DLBCL and p‐DLBCL as a whole. Although p‐DLBCL had superior OS in comparison with t‐DLBCL, the difference was small (OS5, 54.3% vs. 49.6%) and had not statistically significant in multivariate analysis. Compared with the dismal prognosis of t‐FL described before the rituximab era, our study confirmed a good prognosis of t‐DLBCL in the rituximab era, which was even comparable to p‐DLBCL in the same timeframe.

We further explored the prognostic factors associated with PTS10 of t‐DLBCL. Initial treatment strategy before HT was a crucial prognostic factor for PTS10. Patients receiving WW (PTS5, 53.0%; median PTS, 73 months) or RT (PTS5, 66.3%; median PTS, not reached) had a significant PTS10 advantage in comparison with patients receiving CT (PTS5, 43.9%; median PTS, 36 months) or CM (PTS5, 41.7%; median PTS, 27 months). This result is consistent with previous studies, in which treatment‐naïve patients before HT had better PTS.^5^, ^7^, ^8^, ^19^ Furthermore, our study shown that patients receiving RT before HT also had a good prognosis, which has rarely been reported rarely in previous studies. After HT, patients usually received treatment regimens similar to induction regimens for p‐DLBCL, with R‐CHOP being the most common. For initial treatment strategy at HT, patients receiving CT (PTS5, 51.6%; median PTS, 69 months) or CM (PTS5, 56.6%; median PTS, 84 months) were associated with better PTS10 in comparison with patients receiving WW (PTS5, 40.5%; median PTS, 18 months) or RT (PTS5, 30.7%; median PTS, 19 months), which was inverse to the result of initial treatment strategy before HT. In addition, older age at HT and advanced stage at HT were also associated with worse PTS10. Gender, grade, extranodal sites before HT and at HT, and stage before HT had no influence on PTS10.

One interesting finding of our study was that t‐DLBCL with early HT (≥ 18 months) (PTS5, 46.9%; median PTS, 41 months) had a significant PTS10 advantage in comparison with patients with late HT (<18 months) (PTS5, 50.5%; median PTS, 64 months). This phenomenon was also observed in previous two reports.^17^, ^20^ One plausible rationale for the observed disparities in PTS between early and late HT is that early HT may represent a more aggressive process, possibly enhanced by the aggressive component that is not yet recognized. Early and late HT may represent different biologic processes that require molecular characterization for further understanding.

For the choice of treatment after HT, autologous stem cell transplantation (ASCT) was widespread recommended prior to the rituximab era.^21^, ^22^ However, the role of ASCT in t‐FL in the rituximab era is debated, as some studies demonstrated a PTS advantage for t‐FL receiving ASCT,^18^, ^23^, ^24^ while others did not.^17^, ^19^ Considering the heterogeneous prognosis of t‐DLBCL, we performed a subgroup analysis according to the reception of initial treatment strategy before HT. Taking t‐DLBCL and p‐DLBCL as a whole, the OS10 of t‐DLBCL receiving RT or WW was comparable to that of p‐DLBCL, while t‐DLBCL receiving CT or CM had significantly worse OS10 than p‐DLBCL. Therefore, we identified a cohort of t‐DLBCL who do relatively well in the rituximab era. At the same time, some studies also identified a favorable subgroup of t‐FL, mainly treatment‐ or anthracycline‐naïve patients before HT, with excellent outcome without ASCT,^8^, ^17^ which is consistent with our study. By combining our and previous studies in the rituximab era, the initial treatment strategy for t‐FL should be tailored and individualized. Our analysis suggested that ASCT should be avoided in t‐DLBCL receiving RT or WW before HT and should be strongly considered in t‐DLBCL receiving CT or CM before HT.

Several limitations exist due the nature of the SEER registry and lack of central review of pathology slides. As no reassurance exists to the quality of pathological review used to label each patient included in the dataset, we aimed to minimize the risk of incorporating patients who were incorrectly diagnosed with t‐DLBCL without a true culprit HT event by incorporating a delay of 2 months between the diagnosis of FL and t‐DLBCL in the registry. The strength of our study is that SEER registries are population‐based, avoiding potential selection bias that could arise from institution‐based studies. In addition, the large size of the cohort and its long‐running nature are also key strengths of this study.

In conclusion, the outcome of t‐DLBCL shown significant improvement in the rituximab era and was even comparable to p‐DLBCL in the same timeframe. In multivariate analysis, initial treatment strategies of CT or CM before HT, initial treatment strategies of RT or WW at HT, older age at HT, advanced stage at HT, and early HT were associated with worse PTS10 of t‐DLBCL. Given the heterogenous prognosis of t‐DLBCL, we did not recommend ASCT for t‐DLBCL receiving WW or RT due to their comparable survival to p‐DLBCL. These results provided important evidences for the outcome and management strategy of t‐DLBCL.

## AUTHOR CONTRIBUTIONS

**Wenshuai Zheng:** Conceptualization (equal); formal analysis (equal); methodology (equal); software (equal); writing – original draft (equal). **Mingjuan Liu:** Data curation (equal); formal analysis (equal). **Lixun Guan:** Data curation (equal); formal analysis (equal). **Shenyu Wang:** Conceptualization (equal); supervision (equal); writing – review and editing (equal).

## FUNDING INFORMATION

No fund support.

## CONFLICT OF INTEREST STATEMENT

The authors declare no conflict of interest.

## Data Availability

The data that support the findings of this study are available in the SEER database.
